# Bridging evidence worlds: policy and practice pathways for integrating clinical trials and real-world data in drug development and HTA

**DOI:** 10.3389/fphar.2026.1786017

**Published:** 2026-03-06

**Authors:** Alexandros Sagkriotis

**Affiliations:** Independent Consultant in Real-World Evidence and Health Data Science, Helios Academy Limited, Where Science Meets Compassion, London, United Kingdom

**Keywords:** health technology assessment, oncology, ophthalmology, personalized medicine, policy integration, real-world evidence, regulatory science

## Abstract

The integration of clinical and real-world evidence represents one of the most critical transformations in modern drug development and health technology assessment (HTA). Despite increasing regulatory openness, however, clinical trial evidence and real-world data (RWD) still operate largely as parallel rather than fully complementary systems. This commentary argues that experimental and observational research are not competing paradigms but two interdependent components of a unified evidence ecosystem, each capturing distinct yet equally essential dimensions of patient reality. Randomized controlled trials (RCTs) typically enrol highly selected populations and are optimised for internal validity, whereas RWD reflect the heterogeneity, comorbidities, and lived experiences of patients in routine clinical practice. Integrating these two evidence streams is therefore fundamental for personalised medicine, where the objective is to generate evidence that is both scientifically rigorous and meaningfully reflective of individual patient needs. This narrative Policy & Practice Commentary synthesises regulatory guidance, methodological literature, and applied case examples to examine how leading regulatorsand HTA agencies, including the U.S. Food and Drug Administration (FDA), European Medicines Agency (EMA), Medicines and Healthcare products Regulatory Agency (MHRA), Pharmaceuticals and Medical Devices Agency (PMDA), and National Institute for Health and Care Excellence (NICE), are developing frameworks to support the use of RWD and real-world evidence (RWE) in regulatory approval and reimbursement decision-making. Areas of convergence and divergence are explored, with particular emphasis on methodological transparency, data provenance, causal inference, and reproducibility as shared quality anchors across jurisdictions. Illustrative case examples from oncology and ophthalmology are used to demonstrate both the opportunities and challenges of hybrid evidence generation. In oncology, RWD are increasingly applied in external and hybrid control arms, pragmatic designs, and post-authorisation safety and effectiveness studies. In ophthalmology, advances in artificial intelligence-enabled image analytics and disease registries illustrate how routinely collected clinical data and patient-reported outcomes can be integrated to inform real-world value assessment. The commentary concludes by proposing actionable policy and practice recommendations to operationalise integrated evidence models, focusing on harmonised governance and methodological standards, transparent reporting and cross-sector collaboration, systematic incorporation of patient-generated data, and incentives for early and lifecycle-oriented RWD generation. By aligning regulatory policy, analytical methodology, and patient-centred design, integrated RWE frameworks can support decisions that are both scientifically robust and genuinely reflective of real-world patient experience.

## 1 Introduction: from parallel evidence streams to integrated decision-making

Over the past 3 decades, drug development and health technology assessment (HTA) have been dominated by a hierarchical evidence paradigm in which randomized controlled trials (RCTs) occupy the apex as the primary—and often exclusive—source of credible evidence for regulatory approval and reimbursement. This hierarchy, commonly represented as the “evidence pyramid,” prioritises randomized and controlled experimental designs over observational and real-world data sources, reflecting their strength in establishing internal validity and causal inference (Burns et al., 2011; Rothwell, 2005). Within this framework, real-world evidence (RWE) has traditionally been positioned lower in the hierarchy, often regarded as supportive or hypothesis-generating rather than decision-defining (Makady et al., 2017a).

This article is a policy and practice commentary rather than a systematic or scoping review. It does not aim to exhaustively identify or catalogue all relevant literature, nor to apply formal inclusion or exclusion criteria. Instead, it draws on representative regulatory guidance, established methodological literature, and applied case examples selected for their relevance to regulatory, HTA, and payer decision-making. The objective is conceptual integration and practical interpretation rather than comprehensive evidence synthesis.This paradigm has delivered substantial therapeutic advances, yet it has also revealed structural limitations that are increasingly misaligned with contemporary healthcare realities. Patients encountered in routine clinical practice are typically older, more comorbid, more heterogeneous, and more socially diverse than those enrolled in pivotal clinical trials, raising persistent concerns about generalisability and external validity (Rothwell, 2005; Akehurst et al., 2017). In response, regulators have begun to explicitly acknowledge representativeness gaps in clinical research. Notably, the United States Food and Drug Administration (U.S. FDA) now requires sponsors to assess and justify the racial and ethnic distribution of trial populations relative to real-world disease epidemiology and to prospectively plan enrolment strategies that better reflect population diversity (U.S. Food and Drug Administration, 2018).

As a result of these discrepancies, evidence generated under controlled experimental conditions often struggles to fully inform real-world clinical practice and policy decision-making, particularly in settings characterised by multimorbidity, long-term treatment use, and health system complexity (Rothwell, 2005; Akehurst et al., 2017).

Real-world data (RWD) are generally defined as data relating to patient health status and healthcare delivery that are routinely collected outside the context of traditional clinical trials, including electronic health records, disease registries, administrative and claims databases, imaging repositories, digital health technologies, and patient-reported outcomes (U.S. Food and Drug Administration, 2018; Makady et al., 2017b; European Medicines Agency, 2024a). RWE refers to the clinical evidence regarding the usage, benefits, and risks of a medical product derived from the analysis of RWD (U.S. Food and Drug Administration, 2018). These data sources have emerged as a complementary means of addressing evidence gaps left by conventional trials, particularly with respect to long-term outcomes, treatment patterns, and patient-centred measures (Akehurst et al., 2017; Berger et al., 2017).

Despite increasing regulatory openness, clinical trials and RWE still often function in parallel rather than as deliberately integrated components of a unified evidence lifecycle. Comparative analyses across regulatory and HTA bodies consistently demonstrate that RWE is more readily accepted for post-authorisation learning than for initial approval or reimbursement decisions, reinforcing fragmentation across the evidence lifecycle (Rothwell, 2005; Akehurst et al., 2017).

This article argues that the perceived dichotomy between experimental and observational evidence is conceptually flawed. Rather than competing paradigms, RCTs and RWE represent two interdependent perspectives on patient reality: RCTs establish internal validity and causal inference under controlled conditions, while RWE contextualises effectiveness, safety, and value within the complexity of routine care (Burns et al., 2011; U.S. Food and Drug Administration, 2018; Hernán and Robins, 2016; Tunis et al., 2003). Bridging these evidence worlds is therefore not merely a technical challenge, but a policy, governance, and cultural one, with direct implications for personalised medicine, regulatory science, and equitable patient access (Makady et al., 2017b; Akehurst et al., 2017).

## 2 Why integration matters: scientific, clinical, and ethical imperatives

### 2.1 Scientific completeness and external validity

RCTs are optimised to maximise internal validity and causal inference, but they are structurally constrained in their ability to capture long-term outcomes, rare adverse events, and treatment effects in underrepresented populations (Kennedy-Martin et al., 2015; Rothwell, 2006). Importantly, trial populations are not separate from real-world populations but represent a highly selected subset drawn from the broader patient population affected by a given condition. Empirical analyses across therapeutic areas consistently demonstrate that only a minority—often approximately one-third—of real-world patients would meet eligibility criteria for participation in pivotal RCTs, primarily due to age, comorbidities, disease severity, or prior treatment exposure (Kennedy-Martin et al., 2015; Rothwell, 2006; Eichler et al., 2011).

These structural exclusions limit the external validity of trial findings and complicate their translation into routine clinical practice. RWE complements randomized evidence by extending evaluation to patients typically excluded from trials, capturing treatment effects across diverse care pathways, longer time horizons, and real-world patterns of use (Makady et al., 2017a; Berger et al., 2017). When analysed using appropriate causal frameworks, RWE enables a more complete characterisation of effectiveness and safety, addressing evidence gaps that cannot be resolved through experimental designs alone (Hernán and Robins, 2016; Franklin and Schneeweiss, 2017).

### 2.2 Clinical relevance and decision-making

Clinical decision-making rarely occurs under the controlled conditions assumed by traditional trial designs. Clinicians and patients must navigate multimorbidity, treatment sequencing (i.e., the order and timing with which multiple therapies are used across disease progression), variable adherence, and evolving standards of care, often in the absence of direct comparative evidence (Tunis et al., 2003; Rothwell, 2006). In this context, reliance on trial evidence alone may provide an incomplete or overly idealised representation of treatment performance.

Integrated evidence frameworks enable more nuanced benefit–risk assessment by combining internally valid causal estimates from trials with externally valid utilisation, adherence, and safety information from routine care, thereby reducing uncertainty about generalisability and long-term outcomes (Berger et al., 2017; Franklin and Schneeweiss, 2017). Such approaches support treatment personalisation by enabling subgroup analyses, assessment of treatment durability, and evaluation of patient-relevant outcomes that are central to real-world decision-making (Institute of Medicine, 2013).

### 2.3 Ethical and patient-centred considerations

Beyond scientific and clinical considerations, evidence integration carries important ethical implications. Evidence-generation strategies that systematically exclude large segments of the patient population risk reinforcing inequities in access, outcomes, and knowledge generation (Akehurst et al., 2017; World Health Organization, 2017). This concern is particularly salient for older adults, patients with multimorbidity, and socially or ethnically underrepresented groups whose lived experiences are often insufficiently reflected in trial evidence.

Integrating patient-generated data, real-world outcomes, and patient perspectives into evidence frameworks supports a more inclusive and ethically grounded model of scientific evaluation. Such integration aligns evidence generation with the principles of personalised medicine and learning health systems, recognising patients not merely as trial participants but as active contributors to knowledge creation and decision-making (Institute of Medicine, 2013; World Health Organization, 2017).

## 3 Regulatory and HTA landscape: convergence and divergence

The regulatory and HTA environment has evolved substantially over the past decade, shifting from a predominantly post-authorisation view of RWE toward more proactive consideration of RWD across the product lifecycle (Sagkriotis, 2025a; Claire et al., 2024). This evolution reflects growing recognition that traditional clinical trial programmes alone are insufficient to address unmet medical need, accelerated development pathways, and the increasing complexity of personalised medicine. Despite this momentum, acceptance of RWE remains uneven across jurisdictions and decision points, reflecting differences in regulatory mandates, evidentiary standards, and risk tolerance (Hogervorst et al., 2022; Sagkriotis, 2025b). For clarity, this commentary uses “regulatory decision-making” to refer to marketing authorisation and labelling decisions, “HTA” to refer to relative-effectiveness and cost-effectiveness assessment, and “payer/coverage” to refer to reimbursement and access decisions.

### 3.1 Regulatory perspectives

Regulatory agencies increasingly acknowledge that randomised clinical trials alone cannot fully address evidentiary needs in rare diseases, small or stratified populations, and settings requiring long-term safety or effectiveness evaluation. In the U.S, FDA`s Real-World Evidence Program formalised expectations for the use of RWE to support regulatory decision-making, initially focusing on safety surveillance and label expansions, but progressively extending to effectiveness questions under defined conditions (U.S. Food and Drug Administration, 2024; U.S. Food and Dru g Administration, 2025). Central to this evolution is an emphasis on clearly defined research questions, data suitability, prespecified study designs, and transparent analytical plans (U.S. Food and Drug Administration, 2017).

International regulatory guidance increasingly converges around the concept that data must be appropriate for the specific decision context. While earlier frameworks often referred to “fit-for-purpose” data to emphasise methodological adequacy, more recent international guidance—including ICH M14—has adopted the term “fit-for-use,” explicitly linking data suitability to the regulatory decision being addressed (International Council for Harmonisation, 2025). In practice, these concepts are now largely convergent, underscoring that methodological rigour and decision relevance are inseparable requirements.

In Europe, the European Medicines Agency (EMA) has pursued a complementary but distinct approach. Initiatives such as the Data Analysis and Real-World Interrogation Network (DARWIN EU) reflect an ambition to create a federated infrastructure capable of supporting regulatory, HTA, and public-health decision-making through structured access to high-quality RWD (European Medicines Agency, 2024b). These efforts align with broader European policy initiatives, including the European Health Data Space, which seeks to enhance secondary use of health data while strengthening governance and interoperability (European Commission, 2025). EMA guidance consistently positions RWE not as a replacement for randomised evidence, but as a means to contextualise trial findings, explore effectiveness in broader populations, and support benefit–risk reassessment over time (Claire et al., 2024; European Medicines Agency, 2024b).

The United Kingdom’s Medicines and Healthcare products Regulatory Agency (MHRA) has emerged as a particularly active proponent of lifecycle evidence integration. The MHRA has explicitly linked RWD use to innovative licensing pathways, international reliance models, and post-authorisation evaluation, while articulating expectations for governance, data access, and methodological transparency (Medicines and Healthca re products Regulatory Agency, 2021; Eichler et al., 2019). Within Europe, Swissmedic has similarly recognised the role of RWD in regulatory decision-making, while maintaining strong requirements for traceability and analytical robustness (Arondekar et al., 2022).

In Japan, the Pharmaceuticals and Medical Devices Agency (PMDA) has adopted a more cautious but increasingly structured approach, recognising the value of registry data and post-marketing studies while emphasising data standardisation and reliability (Lin et al., 2025). Beyond these major jurisdictions, regulators in Canada, Australia, South Korea, China, Saudi Arabia, and Brazil have now issued formal guidance or pilot frameworks on the use of RWD and RWE, signalling broad global convergence in principle despite continued operational divergence (Zwarenstein et al., 2008; Hernán et al., 2016; Björnsson et al., 2020; Viceconti et al., 2016).

Across regulatory authorities, a common pattern is evident: RWE is considered acceptable when the research question is clearly defined, data provenance is transparent, potential biases are explicitly addressed, and analytical decisions are reproducible (Sagkriotis, 2025a; Claire et al., 2024; International Council for Harmonisation, 2025).

### 3.2 HTA and payer perspectives

HTA bodies have historically been more conservative than regulators in their acceptance of RWE, reflecting their responsibility to inform reimbursement, pricing, and resource-allocation decisions. Nevertheless, a gradual shift is underway. Agencies such as the National Institute for Health and Care Excellence (NICE) now explicitly reference RWE as a component of relative-effectiveness assessment, particularly in areas characterised by clinical uncertainty, rare diseases, and immature trial evidence (El Emam et al., 2020). In Canada, alignment between regulatory and HTA expectations is increasingly evident through coordinated guidance from Health Canada and the Canadian Agency for Drugs and Technologies in Health (CADTH) (Zwarenstein et al., 2008).

In practice, HTA bodies most commonly accept RWE to complement, rather than replace, randomised evidence. Typical use cases include informing long-term outcomes, treatment sequencing, adherence patterns, and health-related quality of life—dimensions that are often insufficiently captured in pivotal trials (Hogervorst et al., 2022; European Medicines Agency, 2025a). From a payer perspective, RWE is most influential when it demonstrates incremental clinical benefit, safety in routine use, patient-relevant outcomes, or value for money, particularly where such evidence materially affects cost-effectiveness estimates (El Emam et al., 2020; European Medicines Agency, 2025a).

Concrete examples of infrastructure-based implementation are well-established at national level. For instance, the monitoring registries operated by the Agenzia Italiana del Farmaco (AIFA; Available from: https://www.aifa.gov.it/registri-farmaci) have been in place for nearly two decades and prospectively collect treatment utilisation, eligibility, and outcome data as conditions of reimbursement for selected high-cost medicines. These registries illustrate how RWD can be embedded directly into coverage decisions and lifecycle reassessment rather than generated retrospectively.

However, HTA acceptance remains contingent on stringent methodological expectations. Persistent concerns regarding confounding, data completeness, and selective reporting—especially in sponsor-generated studies—have contributed to a paradoxical situation in which the same dataset may be acceptable for regulatory learning but insufficient for reimbursement decisions (Hogervorst et al., 2022; Sagkriotis, 2025b). This misalignment continues to limit the routine integration of hybrid evidence across the regulatory–HTA interface.

### 3.3 Areas of convergence and remaining friction

Despite jurisdictional differences, areas of convergence are increasingly apparent. Regulators and HTA bodies now broadly align on core quality principles, including clear data provenance, transparent study protocols, reproducibility of analyses, and explicit communication of uncertainty (Claire et al., 2024; International Council for Harmonisation, 2025; European Medicines Agency, 2025a). There is also growing consensus that early, cross-stakeholder dialogue involving regulators, HTA agencies, and sponsors is essential to ensure that RWD generation is prospective, decision-relevant, and methodologically robust (European Health Data & Evidence Network EHDEN, 2018).

Nevertheless, important frictions remain. Regulatory and HTA guidance is not yet harmonised, and differences in evidentiary thresholds, data access, and incentives for early RWD investment continue to fragment the evidence landscape (Sagkriotis, 2025a; Sagkriotis, 2025b). Comparative policy analyses highlight that while high-level principles converge, operational implementation remains inconsistent across jurisdictions, underscoring the need for coordinated policy action to align expectations across the regulatory–HTA continuum (Sagkriotis, 2025a; Claire et al., 2024).

### 3.4 Data protection and implementation constraints in Europe

A persistent barrier to systematic RWD use within the European Union is the interpretation of the General Data Protection Regulation (GDPR). Although GDPR permits secondary use of health data for scientific and public-health purposes, heterogeneous national interpretations, local ethics requirements, and contractual restrictions frequently delay or prevent cross-border analyses. These operational frictions affect timeliness, scalability, and reproducibility of multi-country RWE studies and represent a non-methodological constraint that can be as limiting as analytical validity. Harmonised governance mechanisms and federated approaches, such as privacy-preserving data networks, are therefore critical to real-world evidence feasibility in practice.

Policy analyses of European RWE implementation have documented that divergent national interpretations of GDPR and heterogeneous data access procedures create operational frictions for multi-country real-world studies, even when methodological standards are aligned (Claire et al., 2024).

## 4 methodological foundations for hybrid evidence generation

The scientific credibility of integrated evidence models rests on methodological rigour, transparency, and governance rather than on the data source alone. As regulatory and HTA bodies expand their openness to RWE, methodological expectations have become clearer: observational data are acceptable when used to address well-defined research questions through study designs and analytical approaches that explicitly consider bias, confounding, and uncertainty (Hogervorst et al., 2022; International Council for Harmonisation, 2025; Eichler et al., 2019).

### 4.1 Study design innovations

Hybrid evidence generation has accelerated the adoption of innovative study designs that seek to combine the strengths of randomised and observational research. External and hybrid control arms represent one of the most visible applications, particularly in settings where randomisation is infeasible, unethical, or impractical. When constructed using prespecified eligibility criteria, aligned endpoints, and robust adjustment strategies, external controls derived from RWD can contextualise single-arm trials and support regulatory and HTA decision-making (Arondekar et al., 2022; Lin et al., 2025).

Pragmatic and registry-based randomised trials further blur the traditional boundary between experimental and real-world research. By embedding randomisation within routine care settings, these designs preserve causal inference while enhancing external validity and operational feasibility. Such approaches are particularly relevant for chronic diseases and long-term outcome evaluation, where conventional explanatory trials may be insufficiently representative (Tunis et al., 2003; Zwarenstein et al., 2008).

Target trial emulation has emerged as a unifying causal framework for observational analyses, explicitly mapping real-world study designs to the hypothetical randomised trial they aim to emulate. This approach enhances transparency by requiring clear articulation of eligibility criteria, treatment strategies, follow-up, and estimands, thereby reducing ambiguity in causal interpretation (Hernán and Robins, 2016; Hernán et al., 2016).

Beyond trial emulation and hybrid control strategies, emerging computational modelling approaches are also being explored to complement real-world data analyses. “Digital twins” refer to computational models that integrate longitudinal clinical data, biological knowledge, and simulation methods to approximate individual patient trajectories; they are not new simulation techniques *per se*, but represent a modern synthesis of modelling and real-world data streams. Importantly, digital twins are discussed here only insofar as they may support hypothesis generation, scenario testing, or methodological development alongside empirical real-world data analyses; they are not presented as a source of effectiveness or comparative evidence for regulatory or HTA decisions (Björnsson et al., 2020; Viceconti et al., 2016).

Synthetic data generation represents a complementary but distinct methodological development. At present, synthetic datasets are primarily used to support method development, privacy-preserving data sharing, and exploratory analyses rather than as direct evidence for regulatory or reimbursement decisions. Regulatory guidance generally emphasises that synthetic data should not replace original patient-level data for decision-critical analyses (El Emam et al., 2020; European Medicines Agency, 2025a).

### 4.2 Analytical approaches and bias mitigation

Advanced analytical methods play a central role in ensuring the credibility of hybrid evidence. Techniques such as propensity score matching, inverse probability weighting, and doubly robust estimation are widely used to address measured confounding, while sensitivity analyses are increasingly expected components of regulatory and HTA submissions (Hernán and Robins, 2016; Franklin and Schneeweiss, 2017; Hogervorst et al., 2022). Importantly, no single method is universally sufficient; analytical choices must be justified in relation to the specific research question, data structure, and decision context (Hogervorst et al., 2022; International Council for Harmonisation, 2025).

Unmeasured confounding and data missingness remain fundamental challenges. Contemporary guidance encourages explicit quantification of uncertainty and transparent discussion of residual bias, recognising that uncertainty is inherent to all forms of evidence generation—including randomised trials—and must be managed rather than ignored (Hernán and Robins, 2016; International Council for Harmonisation, 2025).

### 4.3 Data quality, governance, and reproducibility

Data quality is a foundational requirement for RWE acceptability, yet quality is increasingly defined in terms of fitness for decision context rather than abstract completeness. Recent international guidance reflects convergence between the concepts of “fit-for-purpose” and “fit-for-use,” emphasising that data must be both methodologically sound and demonstrably appropriate for the specific regulatory or HTA decision (International Council for Harmonisation, 2025; Eichler et al., 2019).

Regulators and HTA bodies therefore focus on data provenance, longitudinal integrity, outcome validity, and traceability of data transformations applied during analysis (U.S. Food and Drug Administration, 2024; European Medicines Agency, 2024b). Federated data networks and common data models—such as those underpinning DARWIN EU and EHDEN—support reproducibility while respecting privacy and local governance constraints (European Medicines Agency, 2024b; European Health Data & Evidence Network EHDEN, 2018).

Equally important is analytical transparency. Preregistration of observational study protocols, version-controlled code, and auditable analytical pipelines are increasingly viewed as expectations rather than aspirational best practices, particularly when analyses inform high-stakes regulatory or reimbursement decisions (von Elm et al., 2007; Benchimol et al., 2015).

### 4.4 Role of artificial intelligence and advanced analytics

Artificial intelligence (AI) and machine-learning techniques offer substantial opportunities to extract value from high-dimensional RWD, including imaging, genomics, and unstructured clinical text. In therapeutic areas such as oncology and ophthalmology, AI-enabled image analytics can generate quantitative endpoints at scale, transforming routinely collected data into analysable outcomes (Björnsson et al., 2020; Beaver and Pazdur, 2022).

However, AI introduces additional methodological and ethical considerations. Algorithmic opacity, bias amplification, and challenges in generalisability necessitate robust validation, explainability, and lifecycle governance. Regulators increasingly emphasise transparency, external validation, and continuous performance monitoring for algorithms used within evidence-generation pipelines (Eichler et al., 2019; Benchimol et al., 2015).

Taken together, these methodological foundations reinforce a central principle: integrating clinical trials and RWD does not relax scientific standards but expands the methodological toolkit to address questions that neither paradigm can adequately answer alone (Hogervorst et al., 2022; International Council for Harmonisation, 2025).

## 5 Illustrative case examples

The practical value of integrating clinical trial data with RWD is most clearly illustrated in therapeutic areas characterised by high uncertainty, rapid innovation, and heterogeneous patient populations. Oncology and ophthalmology exemplify both the opportunities and methodological complexities of hybrid evidence generation, offering complementary perspectives on how integrated evidence can inform regulatory, HTA, and clinical decision-making across the product lifecycle (Hogervorst et al., 2022; Arondekar et al., 2022).

### 5.1 Oncology: complexity, urgency, and adaptive evidence generation

Oncology has emerged as a leading test case for hybrid evidence approaches due to the convergence of unmet medical need, accelerated regulatory pathways, and increasingly stratified patient populations. Randomised controlled trials in oncology are frequently constrained by small sample sizes, narrow eligibility criteria, and rapidly evolving standards of care, particularly in rare cancers and biomarker-defined subgroups (Beaver and Pazdur, 2022; Gyawali et al., 2020).

To enable timely trial completion, many oncology trials rely on surrogate endpoints such as progression-free survival (PFS) rather than overall survival (OS). However, the strength and consistency of the association between PFS and OS varies substantially across cancer types, disease stages, and therapeutic classes, limiting the generalisability of surrogate-based conclusions (Belin et al., 2020; Booth et al., 2023). These limitations heighten uncertainty at the time of approval and reimbursement, creating a need for complementary evidence sources.

In this context, RWD has been increasingly used to augment clinical trial evidence across multiple decision points. External control arms derived from high-quality registries and electronic health record data have supported the interpretation of single-arm trials, particularly where randomisation is infeasible or ethically challenging. When constructed using aligned eligibility criteria, harmonised endpoints, and transparent analytical methods, such external controls can provide meaningful contextualisation of treatment effects while preserving scientific credibility (Arondekar et al., 2022; Lin et al., 2025).

Beyond initial approval, oncology illustrates the lifecycle value of real-world evidence. Post-authorisation analyses of treatment sequencing, real-world progression endpoints, and long-term survival outcomes extend beyond the temporal limits of pivotal trials and have informed regulatory reassessments, HTA reviews, and clinical guideline updates (European Medicines Agency, 2024b; Beaver and Pazdur, 2022). At the same time, oncology highlights persistent methodological challenges, including confounding by indication, incomplete capture of disease progression, and heterogeneity in diagnostic and treatment practices. These limitations reinforce the importance of prespecified protocols, sensitivity analyses, and cautious interpretation, underscoring that integration enhances—but does not eliminate—uncertainty (Hogervorst et al., 2022; International Council for Harmonisation, 2025).

In practice, regulatory and HTA decisions do not always align. Several oncology products have received accelerated or conditional approval supported by single-arm trials contextualised with external or registry-based controls, while HTA bodies subsequently requested additional comparative or survival evidence before granting full reimbursement (Gyawali et al., 2020; Belin et al., 2020; Booth et al., 2023). Such divergences illustrate how identical real-world data may be considered sufficient for regulatory plausibility yet insufficient for cost-effectiveness modelling, underscoring the importance of early joint evidence planning.

Taken together, these applications demonstrate how integrated trial and real-world evidence can reduce decision uncertainty by extending inference beyond trial populations and time horizons, directly informing regulatory reassessment, HTA deliberation, and clinical guideline development.

### 5.2 Ophthalmology: registries, imaging, and patient-centred outcomes

Ophthalmology provides a contrasting but equally instructive model for integrated evidence generation. Many ophthalmic diseases are chronic, slowly progressive, and managed predominantly in outpatient settings, creating favourable conditions for longitudinal data collection through registries and routine clinical care (Daien et al., 2021).

Disease-specific registries in ophthalmology have enabled systematic linkage of clinical outcomes, imaging data, and patient-reported measures over extended follow-up periods. These registries complement trial evidence by capturing treatment durability, real-world adherence, and functional outcomes that are central to patient experience but often under-represented in controlled studies (Sagkriotis et al., 2021; Shellvarajah et al., 2025; Chakravarthy et al., 2021).

A defining feature of ophthalmology is the increasing role of imaging-based RWD. Advances in AI and image analytics have enabled quantitative assessment of anatomical and functional biomarkers at scale, transforming routinely collected retinal images into analysable endpoints. Pioneering work in this field—led by academic centres including Vienna, University College London, Sydney, and Zurich—demonstrates how AI-derived measures can augment trial data and enhance understanding of disease progression and treatment response in real-world populations (Schmidt-Erfurth et al., 2018; De Fauw et al., 2018; Yim et al., 2020; Spooner et al., 2025).

However, ophthalmology also underscores important governance and standardisation challenges. Variability in imaging protocols, device calibration, and annotation practices can affect data comparability and generalisability. Addressing these challenges requires rigorous validation, harmonisation of data standards, and transparent reporting—principles that align closely with broader methodological expectations for hybrid evidence generation (von Elm et al., 2007; Benchimol et al., 2015).

For example, large registry–imaging linkages have been used to evaluate durability of anti-VEGF regimens and treatment interval optimisation beyond the time horizon of pivotal trials, informing both post-authorisation safety surveillance and payer reassessment of real-world effectiveness (Shellvarajah et al., 2025; Chakravarthy et al., 2021; Spooner et al., 2025).

These examples illustrate how registry-based data and AI-derived imaging endpoints can generate quantifiable, decision-relevant outcomes at scale, supporting post-authorisation safety evaluation and payer reassessment of real-world effectiveness.

### 5.3 Cross-cutting lessons from case examples

Taken together, oncology and ophthalmology demonstrate that there is no single model for integrating clinical trials and RWD. Instead, effective hybrid evidence generation depends on aligning methodological choices with disease context, decision needs, and data availability. Across both therapeutic areas, common enablers emerge: early planning, cross-stakeholder collaboration, transparent governance, and sustained investment in data infrastructure (Claire et al., 2024; European Medicines Agency, 2024b).

These case examples reinforce the central argument of this article: integrated evidence is most impactful when it is designed prospectively, interpreted cautiously, and embedded within a lifecycle framework that recognises both scientific rigour and patient reality (Sagkriotis, 2025a; Hogervorst et al., 2022).

## 6 Policy and practice recommendations

Translating the integration of clinical trials and RWD into routine regulatory and HTA practice requires more than methodological capability. It demands coordinated policy action, shared governance principles, and aligned incentives across the healthcare ecosystem. Building on the preceding analysis, this section proposes actionable recommendations across four interdependent domains.

### 6.1 Governance and methodological harmonisation

Regulatory authorities and HTA bodies should continue to converge on shared foundational principles for real-world evidence (RWE) acceptability, while allowing flexibility in implementation according to decision context. Clear articulation of acceptable study designs, estimands, and analytical approaches—developed through joint scientific advice and early dialogue—can reduce duplication and misalignment across regulatory and reimbursement pathways (European Medicines Agency and EUnetHTA 21 Consortium, 2022; Németh et al., 2023).

Harmonisation should focus on principles rather than prescriptive methods. Emphasising transparency, decision-context suitability (“fit-for-use”), and explicit uncertainty assessment allows methodological innovation while preserving scientific rigour, consistent with international guidance on non-interventional RWD studies (International Council for Harmonisation of Technical Requirements for Pharmaceuticals for Human Use ICH, 2025). International collaboration among agencies can further support consistency, particularly for global development programmes and rare diseases where evidence generation is inherently constrained (Németh et al., 2023).

In this context, recent policy analyses suggest that regulatory guidance remains insufficiently harmonised across jurisdictions and that pragmatic reliance pathways and lifecycle evidence pilots may help align expectations—provided governance and transparency standards are clearly defined (Claire et al., 2024; Németh et al., 2023).

### 6.2 Transparent reporting and reproducibility standards

The credibility of integrated evidence depends fundamentally on transparent reporting and reproducibility. Standardised reporting expectations for RWE studies—aligned with trial reporting standards and supported by regulator-endorsed methodological guidance—should be consistently required across regulatory submissions and HTA dossiers (European Network of Centres for Pharmacoepidemiology and Pharmacovigilance ENCePP, 2023).

Preregistration of observational study protocols, public availability of analysis plans, and use of version-controlled code repositories can substantially enhance trust in hybrid evidence. While these practices increase upfront effort, they reduce downstream uncertainty and facilitate independent scrutiny, ultimately strengthening decision confidence (European Network of Centres for Pharmacoepidemiology and Pharmacovigilance ENCePP, 2023).

To further enhance credibility, decision-critical post-launch RWE—particularly studies intended to support label extensions, reimbursement reassessments, or major benefit–risk decisions—should preferentially be conducted through independently governed RWD hubs, academic consortia, or regulator-endorsed networks rather than being fully sponsor-controlled. This proposal aligns with concerns raised in your governance analysis of non-submission RWE, where inconsistent oversight and selective transparency can undermine confidence even when methodological standards are met (Sagkriotis, 2025b).

### 6.3 Systematic integration of patient-generated data

To realise the promise of personalised medicine, patient-generated data—including patient-reported outcomes, digital health data, and real-world functional measures—must be embedded more systematically within evidence frameworks. Regulators and HTA bodies should provide clearer guidance on how such data can inform benefit–risk assessment, comparative effectiveness, and value evaluation, particularly when conventional trial endpoints fail to reflect outcomes that matter most to patients.

In addition to structured patient-reported outcomes, ethically governed social media listening has emerged as a complementary method for understanding lived experience, unmet needs, and treatment burden—especially in oncology and advanced disease settings. Published social media listening studies in oncology and haematology demonstrate how systematic social media analyses can generate real-world insights that are rarely captured in trials or administrative datasets (Rodrigues et al., 2022; Mazza et al., 2022; Chauhan et al., 2022; Perić et al., 2023).

Meaningful patient involvement in study design, endpoint selection, and interpretation is essential to ensure relevance and ethical legitimacy, moving beyond tokenistic consultation toward sustained partnership models that recognise patients as contributors to evidence generation rather than passive data sources.

### 6.4 Incentives for early and lifecycle-oriented RWD use

Current incentive structures often encourage retrospective and fragmented RWD use, limiting decision impact. Policy mechanisms that promote early planning of RWD generation—aligned with clinical development programmes—can enhance evidence coherence. Early scientific advice involving regulators, HTA bodies, and sponsors should explicitly address how RWD will complement trial evidence across the product lifecycle (European Medicines Agency and EUnetHTA 21 Consortium, 2022; Németh et al., 2023).

Post-authorisation commitments, adaptive reimbursement models, and outcomes-based agreements offer additional opportunities to embed RWE into ongoing evaluation, but require proportionate governance and sustainable data infrastructure.

A pragmatic implementation model would involve predefined post-launch RWD networks or hubs generating evidence for a defined period (e.g., the first 3–5 years after launch), until sufficient confidence in safety, effectiveness, and real-world use is established. This builds on established EU post-authorisation study expectations and U.S. active surveillance infrastructure (European Medicines Agency, 2017; European Medicines Agency, 2025b; U.S. Food and Drug Administration, 2022), while recognising staggered launches across countries necessitate flexible, federated implementation.

### 6.5 Building a sustainable evidence ecosystem

Successful integration depends on sustained investment in data infrastructure, analytical capability, and cross-sector collaboration. Federated data networks and common data models support scalability while respecting local governance and privacy constraints. Capacity-building within regulatory agencies, HTA bodies, and clinical communities is equally critical to ensure integrated evidence is interpreted and applied consistently.

Proposal (post-launch predefined networks): We propose that, for a defined period following market authorisation (e.g., the first three to 5 years, depending on indication, novelty, and residual uncertainty), sponsors and health systems should generate real-world evidence through pre-specified networks or hubs, such as federated data partners, disease registries, or active surveillance infrastructures, operating under agreed protocols, endpoints, and governance arrangements. Such models are consistent with established regulatory expectations for post-authorisation evidence generation in Europe and with active surveillance frameworks in the United States, including the FDA Sentinel System (European Medicines Agency, 2017; European Medicines Agency, 2025b; U.S. Food and Drug Administration, 2022; U.S. Food and Drug Administration, 2024).

Once sufficient confidence in real-world safety, effectiveness, and patterns of use has been established—calibrated to the product’s risk profile and evidence maturity—the intensity of evidence generation can be proportionately reduced. However, variation in launch timing across countries complicates synchronised implementation, reinforcing the need for scalable, federated approaches rather than single-country or sponsor-bound solutions (European Medicines Agency, 2017; U.S. Food and Drug Administration, 2022).

Together, these recommendations emphasise that integration of clinical trials and real-world data is not a one-time methodological exercise, but a continuous process of alignment across policy, practice, and patient experience (Institute of Medicine, 2013; World Health Organization, 2017).

## 7 Discussion: toward an integrated evidence ecosystem

This commentary builds on the preceding sections to integrate regulatory guidance, methodological foundations, and therapeutic case examples into a coherent model for lifecycle evidence integration. Rather than revisiting scope or taxonomy, the discussion focuses on governance, implementation, and ethical implications of sustained hybrid evidence generation.

This article has examined the integration of clinical trial evidence and RWD as a structural transformation in drug development and HTA, rather than as a transient methodological trend. Across regulatory policy, analytical innovation, and therapeutic case examples, a consistent message emerges: the limitations of traditional evidence hierarchies are increasingly exposed by the complexity of modern medicine, while hybrid evidence models offer a pathway toward more complete, decision-relevant evaluation (Burns et al., 2011; Rothwell, 2005; Sagkriotis, 2025a; Claire et al., 2024).

Discourse surrounding real-world evidence (RWE) has often been polarised, framed in terms of substitution *versus* preservation of randomised evidence. Such framing obscures the more substantive issue—namely, that no single evidence source is sufficient to address the full spectrum of regulatory, clinical, and societal questions associated with contemporary therapies. The challenge, therefore, is not whether to integrate RWD, but how to do so in a manner that preserves scientific integrity while enhancing relevance across the evidence lifecycle (Makady et al., 2017a; Franklin and Schneeweiss, 2017; Hogervorst et al., 2022).

A central insight from this commentary is that integration is fundamentally a governance challenge rather than a methodological one. While analytical tools for hybrid evidence generation—such as target trial emulation, external control arms, and pragmatic designs—are increasingly mature, their impact remains constrained by fragmented policy expectations, misaligned incentives, and variable institutional capacity (International Council for Harmonisation, 2025; Eichler et al., 2019; Hernán et al., 2016). Where early dialogue, transparent protocols, and shared quality principles are in place, integrated evidence has demonstrably informed regulatory reassessment, HTA deliberation, and clinical guidance (U.S. Food and Drug Administration, 2024; European Medicines Agency, 2024b; European Medicines Agency and EUnetHTA 21 Consortium, 2022). Where such alignment is absent, RWD risks being perceived as opportunistic or insufficiently credible, regardless of analytical sophistication (Sagkriotis, 2025b; European Network of Centres for Pharmacoepidemiology and Pharmacovigilance ENCePP, 2023).

The oncology and ophthalmology case examples further illustrate that evidence integration is inherently context dependent. Disease trajectory, endpoint maturity, outcome measurability, data availability, and patient experience all shape the appropriate balance between experimental and observational evidence (Arondekar et al., 2022; Beaver and Pazdur, 2022; Gyawali et al., 2020; Belin et al., 2020; Daien et al., 2021; Sagkriotis et al., 2021; Shellvarajah et al., 2025; Chakravarthy et al., 2021; Schmidt-Erfurth et al., 2018; De Fauw et al., 2018; Yim et al., 2020; Spooner et al., 2025). Recognising this heterogeneity is essential to avoid one-size-fits-all frameworks that risk undermining both innovation and trust. Instead, flexible, decision-focused integration strategies—anchored in disease context and uncertainty tolerance—are required.

Finally, the discussion returns to the ethical dimension of evidence integration. As healthcare systems move toward personalised medicine, evidence frameworks must evolve to reflect the diversity and lived realities of patients rather than an idealised “average” trial participant (Institute of Medicine, 2013; World Health Organization, 2017; Mazza et al., 2022). Integrating real-world outcomes and patient-generated data is therefore not only a scientific necessity but a moral one, ensuring that regulatory and reimbursement decisions remain grounded in human relevance as well as statistical validity (Sagkriotis, 2025a; Rodrigues et al., 2022; Mazza et al., 2022; Chauhan et al., 2022; Perić et al., 2023).

## 8 Conclusion

The integration of clinical trial evidence and real-world data represents a necessary evolution in how medicines are evaluated, regulated, and reimbursed in contemporary healthcare systems. As this article has demonstrated, randomised and observational evidence are not competing paradigms but complementary components of a more complete and patient-relevant evidence ecosystem (Burns et al., 2011; Makady et al., 2017a; Franklin and Schneeweiss, 2017; Sagkriotis, 2025a).

Regulatory and HTA bodies have made meaningful progress in articulating pathways for the use of real-world evidence, yet routine and consistent integration remains constrained by fragmented governance, variable methodological expectations, and misaligned incentives (Claire et al., 2024; Hogervorst et al., 2022; Sagkriotis, 2025b). Addressing these challenges requires early and sustained dialogue across stakeholders, transparent and reproducible analytical practices, and policy frameworks that support lifecycle-oriented evidence generation rather than retrospective and opportunistic data use (International Council for Harmonisation, 2025; European Medicines Agency and EUnetHTA 21 Consortium, 2022).

Therapeutic areas such as oncology and ophthalmology illustrate that integrated evidence can meaningfully inform regulatory, reimbursement, and clinical decisions when it is designed with context, uncertainty, and patient experience in mind (Arondekar et al., 2022; Beaver and Pazdur, 2022; Gyawali et al., 2020; Belin et al., 2020; Daien et al., 2021; Sagkriotis et al., 2021; Shellvarajah et al., 2025; Chakravarthy et al., 2021; Schmidt-Erfurth et al., 2018; De Fauw et al., 2018; Yim et al., 2020; Spooner et al., 2025). These examples also underscore that integration is not a one-size-fits-all solution, but a flexible approach that must adapt to disease characteristics, data availability, and decision needs.

Ultimately, bridging evidence worlds is not solely a technical exercise. It represents a commitment to aligning scientific rigour with clinical reality and ethical responsibility. By embedding integration into policy, practice, and organisational culture, hybrid evidence models can support decisions that are not only statistically robust, but also genuinely reflective of the patients they are intended to serve (Institute of Medicine, 2013; World Health Organization, 2017; Sagkriotis, 2025a; Mazza et al., 2022).
